# *Panax quinquefolium* L. Ginsenosides from Hairy Root Cultures and Their Clones Exert Cytotoxic, Genotoxic and Pro-Apoptotic Activity towards Human Colon Adenocarcinoma Cell Line Caco-2

**DOI:** 10.3390/molecules25092262

**Published:** 2020-05-11

**Authors:** Ewa Kochan, Adriana Nowak, Małgorzata Zakłos-Szyda, Daria Szczuka, Grażyna Szymańska, Ilona Motyl

**Affiliations:** 1 Pharmaceutical Biotechnology Department, Medical University of Lodz, Muszyńskiego 1, 90-151 Lodz, Poland; grazyna.szymanska@umed.lodz.pl; 2 Department of Environmental Biotechnology, Lodz University of Technology, Wólczańska 171/173, 90-924 Lodz, Poland; 203190@edu.p.lodz.pl (D.S.); ilona.motyl@p.lodz.pl (I.M.); 3 Institute of Molecular and Industrial Biotechnology, Lodz University of Technology, Stefanowskiego 4/10, 90-924 Lodz, Poland; malgorzata.zaklos-szyda@p.lodz.pl

**Keywords:** *Panax quinquefolium* L., hairy roots, ginsenosides, cytotoxicity, genotoxicity, apoptosis, necrosis, mitochondrial membrane potential, ATP, Caco-2

## Abstract

American ginseng, *Panax quinquefolium* (L.), is traditionally used in folk medicine. It exhibits a range of anti-inflammatory, hepatoprotective, anti-diabetic, anti-obesity, anti-hyperlipidemic and anti-carcinogenic effects. Its main components are ginsenosides, also known as panaxosides or triterpene saponins. In order to obtain high yields of ginsenosides, different methods of controlled production are involved, i.e., with hairy root cultures. However, they are still employed under in vitro conditions. Our studies revealed that hairy root cultures subjected to an elicitation process can be considered as a potent source of ginsenosides. The present study examines the biological activity of ginseng hairy root cultures against the Caco-2 human adenocarcinoma cell line. Among our six different clones of *P. quinquefolium* hairy roots, extracts B and B*e* (treated with elicitor) were the strongest inhibitors of the cellular metabolic activity. While all extracts induced DNA damage, B and B*e* also generated reactive oxygen species (ROS) in a concentration-dependent manner, which was correlated with the depletion of the mitochondrial membrane potential and induction of apoptosis. These findings indicate that further research concerning *P. quinquefolium* hairy root cultures should focus on the activity of rare ginsenosides and other biologically active compound profiles (i.e., phenolic compounds).

## 1. Introduction

Plants with healing properties have been applied in medicine and folk herbal practices for centuries. One species used for hundreds of years for its therapeutic properties is American ginseng, known as *Panax quinquefolium (L.) Alph. Wood (synonym Panax quinquefolius (L.), Aralia quinquefolia (L.) Decne. & Planch, Ginseng quinquefolium (L.) Alph. Wood, Panax americanus (Raf.) Raf., Panax cuneatus Raf.)* [1]. Ginseng roots and their extracts are used in pharmacy and cosmetics and as functional foods or dietary supplements. In 2015, the American Council for Responsible Nutrition reported that 31% of the consumers used herbal supplements to cope with various health problems and ginseng was ranked fourth among leading dietary supplements. It is also added to beverages, smoothies or green drinks to enhance their health beneficial properties.

Ginseng exhibits various anti-inflammatory, hepatoprotective, anti-diabetic, anti-obesity, anti-hyperlipidemic and anti-carcinogenic effects, as well as a tonic effect [2]. As the major bioactive ingredients of ginseng are ginsenosides, also known as panaxosides or triterpene saponins, the extracts used in industry are usually standardized for the ginsenoside content. Ginsenosides are glycosidic compounds consisting of a non-sugar aglycone part and either single or multiple sugar chains. Three types of aglycones can be distinguished: tetracyclic aglycones such as dammaran (the most important are 20 (*S*)-protopanaxadiol and 20 (*S*)-protopanaxatriol), pentacyclic aglycones such as oleanolic acid and tetracyclic aglycones such as ocotillol. The sugar part of the saponin most often includes hexoses (glucose, galactose), 6-deoxyhexoses (furanose, rhamnose), pentoses (arabinose, xylose) or uronic acids (i.e., glucuronic acid); they usually have cyclic structures and form semi-acetic bonds with an aglycone.

Most ginsenosides are glycoside derivatives of dammaran consisting of 17 carbon atoms in a four-ring structure with various sugar residues attached to the positions C-3 and C-20 [3]. Over 30 of the so-called main ginsenosides were identified. They can be divided into two types: 20 (*S*)-protopanoxadiol (PPD) derivatives and 20 (*S*)-protopanoxatriol (PPT) derivatives. The PPD derivatives include such metabolites as Rb1, Rb2, Rb3, Rc and Rd. They are denoted as the Rb group of ginsenosides. Other ginseng saponins belonging to the PPT derivatives, such as Re, Rf, Rg1, Rg2 and Rh1, are known as the Rg group of ginsenosides [3]. The dammaran-type metabolites, such as the saponins Rh2, Rh3, Rh4, Rg2, Rg3, Rg5 and Rk1, are rare and are referred to as minor ginsenosides. Several reports note that they are naturally present in trace amounts or they are not detected at all. In raw plant material, their level can be altered, enhanced or enriched using several different techniques such as steaming, puffing, fermentation and high-temperature/pressure treatments [4,5,6,7].

Ginsenoside F11 (24-*R*-pseudoginsenoside) is an ocotillol-type saponin, while ginsenoside Ro is pentacyclic [8]. Bioactive phytochemicals from *P. quinquefolium*, as well as their chemical structure, biochemistry, pharmacological and biological activity, have been thoroughly discussed in a recent review [3].

Previously, the raw material for the production of medicinal ginseng products was obtained from natural sources; however, due to extensive exploitation resulting in ginseng being entered into the “Red Book of Endangered Species” in 1972, new attempts have been made to cultivate ginseng under natural conditions or field conditions. Currently, the high demand for and high prices of ginseng root (from 20 to 1105 USD per kilogram), as well as the inability to obtain this raw material from natural sources, have caused an increase in field ginseng cultivation. However, soil cultivation is very labour-intensive, and at least three or four years is needed to obtain valuable raw material. In addition, due to the need for agro technology and prophylactic plant protection treatments (ginseng is extremely susceptible to fungal diseases and pests), the process is expensive [9,10,11]. Therefore, in order to obtain plant ginsenosides with high yields, different controlled productions based on the use of hairy root cultures in vitro are under investigation.

Such cultivation methods may be an effective way to obtain valuable secondary metabolites for field crops. Hairy root cultures possess advantages over other cultivation methods [12]. They are characterized by rapid growth (only 28 days) without the need for supplementation with additional phytohormones, which allows a large amount of biomass to be produced in a relatively short time. In addition, they are genetically stable and no drastic decline in metabolite accumulation is observed as the root line grows older. They are distinguished by plagiatropism and a lack of geotropism. Hairy root cultures involve the production of numerous lateral roots with an increased root hair zone, diversified cell structure and structural integration of tissues, which plays a crucial role in the normal course of metabolic processes. This is especially important considering that some metabolites are synthesized only in specialized organs and usually only appear in the above-ground parts of plants. In addition, the approach also offers a relative ease in changing the scale of production, which further increases their value as potential “producers” of desired compounds [13].

Our previous investigation indicated that hairy root cultures of *P. quinquefolium* could serve as an alternative source of plant material for industrial use, as they readily accumulate ginsenosides in the same or higher amounts than traditionally cultivated roots [14,15]. However, knowledge of the active compound content must be supplemented with an understanding of the biological properties of these cultures.

In the present studies, three clones of hairy root cultures of *P. quinquefolium* (labelled A, B and G) were examined for their biological effects; these were either subjected to a methyl jasmonate elicitation or not. The novelty of this investigation lies in the fact that it examines the genotoxic and cytotoxic potency of the tested extracts towards the Caco-2 human colon adenocarcinoma cell line using a comet assay (measuring DNA damage) and two commercial cytotoxicity assays: MTT (3-(4,5-dimethylthiazolyl-2)-2,5-diphenyltetrazolium bromide) and PrestoBlue. In addition, the cells were subjected to a microscope observation to identify any morphological changes. A clonogenic assay was performed to measure the proliferative capacity of cells after treatment with the *P. quinquefolium* extracts. To investigate the potential of the extracts as inducers of apoptosis/necrosis, the intracellular ATP level, mitochondrial membrane potential and intracellular oxidative stress were also investigated.

## 2. Results and Discussion

### 2.1. Ginsenoside Content in Studied Clones of Hairy Root Cultures of P. quinquefolium

Three clones of *P. quinquefolium* hairy roots (A, B and G) were examined to determine their biological properties. Transformation was confirmed by a PCR analysis [16]. This analysis confirmed that the *rol B* and *rol C* genes from the Ri plasmid of *A. rhizogenes* became integrated with the genome of the *P. quinquefolium* hairy roots and thus indicated the presence of integrated T-DNA in the hairy root cultures.

**Figure 1:**
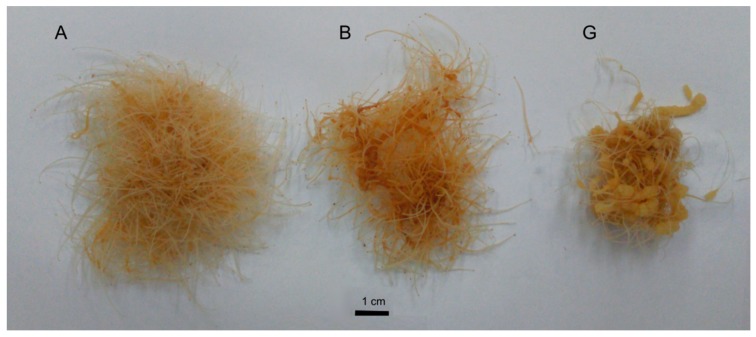
Morphology of A, B and G clones of hairy root cultures of *P. quinquefolium* after 28-days cultivation.

The studied clones differed in terms of morphology (Figure 1) and content of active compounds–ginsenosides (Table 1).

Line A demonstrated the morphology typical for hairy roots, with thin roots of a light-yellow colour. The roots from line B were also thin; however, their oldest part became brown. Additionally, they achieved a lower biomass production than those of clone A. The roots of clone G were partially thicker and had a callus-like appearance. The extracts in which the level of ginsenosides was examined were derived from the roots cultures that did not undergo an elicitation process (A, B and G), as well as those subjected to a MeJA elicitation (A*e*, B*e* and G*e*). The hairy roots that underwent elicitation contained more saponins than those untreated with MeJA (Table 1).

The highest levels of total ginsenosides were determined in clone A and A*e* (17.04 and 34.96 mg/g d.w., respectively). Both hairy root cultures were the richest in their Rb saponin content, expressed as the sum of Rb1, Rb2, Rb3, Rc and Rd; however, the protopanaxadiol derivatives content was 2.4-fold higher in A*e* than clone A. In addition, the levels of the Rb group saponins also increased more than 4-fold in B*e* and 4.7-fold in G*e*, i.e., after stimulation with MeJa, compared with the non-treated samples.

Slightly different findings were obtained for the Rg ginsenosides (expressed as sum of Rg1 and Re). Among the untreated cultures, clone A was found to express the greatest amount of protopanaxatriol derivatives. Among the treated cultures, clone G*e* accumulated higher amounts of the Rg group saponins than A*e* and B*e*. Additionally, clone A*e* demonstrated lower Rg1 + Re than A.

An analysis of the individual saponins showed that the quantitatively dominant compounds were Rb1 and Rc (clone A), Rc and Rb1 (clone B) or Rb1 and Rg1 (clone G) in the cultures not subjected to elicitation. Further, metabolites Rb1 and Rd were found to predominate in all cultures (A*e*, B*e*, G*e*) after the MeJA treatment. Our results demonstrate that ginsenoside profiles varied significantly among the hairy root clones both with regard to the type of clone and elicitation status. The untreated clones demonstrated the following ginsenoside profiles: Rb1 > Rc > Re > Rd > Rb2/Rg1 > Rb3 for clone A, Rc > Rb1 > Rg1 > Re > Rd > Rb2 > Rb3 for clone B and Rb1 > Rg1 > Rc > Re > Rd > Rb2 > Rb3 for clone G. In contrast, the elicited cultures demonstrated quite different profiles: Rd > Rb1 > Rc > Rb2 > Rb3 > Re > Rg1 for A*e*, Rb1 > Rd > Rc > Rg1 > Re > Rb2 > Rb3 for B*e* and Rb1 > Rd > Rg1 > Rc > Re > Rb2 > Rb3 for G*e* (Figure S1).

Elicitation, i.e., the treatment of a culture with an elicitor, is one of the most frequently applied methods used to increase the secondary metabolite production in in vitro cultures. It is based on the subjecting of the studied culture to the activity of the elicitor. An elicitor is a chemical compound that can enhance the synthesis of biologically active compounds in plants by causing defensive reactions. These compounds can be important ingredients from a commercial point of view [17]. In this case, MeJA was used as the elicitor. Saponin production increased twofold in the A*e* line and threefold in the B*e*/G*e* lines of the *P. quinquefolium* hairy roots compared with the non-elicited roots. This observation is not surprising considering previous studies [15,18] indicating that MeJA boosted the expression of genes coding key enzymes involved in ginsenoside biosynthesis; more specifically, 250 µM MeJA was found to be the most optimal concentration for an effective ginsenoside accumulation [15]. These observations are analogous to in vivo conditions where environmental factors very often strongly influence the production of secondary metabolites; hence, exposure to exogenous methyl jasmonate also influences the ginsenoside production.

The influence of in vitro elicitation on the content of the secondary metabolites in the hairy root clones was also examined for *Gentiana cruciata* or *Psammosilene tunicoides* [19,20]. In the present study, the A, B and G root lines not only demonstrated differences in the ginseng saponin production but they were characterized by different morphologies. These disparities can be connected with the random integration of T-DNA into the Ri plasmid in plant tissue. Previous research indicated that even following a successful transformation, the length and copy number of T-DNA inserted into a plant cell varies, resulting in variation in the morphology, genetics, physiology and biochemistry of the resulting clone, i.e., with a different metabolic state and the capacity for the synthesis of secondary metabolites [21,22]. Additionally, some reports on plants from the *Araliaceae* (for which *P. quinquefolium* belongs), *Solanaceae*, *Rubiaceae*, *Vitaceae* or *Rosaceae* indicate that the *rol A*, *rol B* and *rol C* oncogenes, included in T-DNA, are capable to modulate plant growth, cell differentiation and be potential activators of secondary metabolism in transformed cells [21,23].

### 2.2. Cytotoxic Activity of P. quinquefolium Extracts

**Figure 2:**
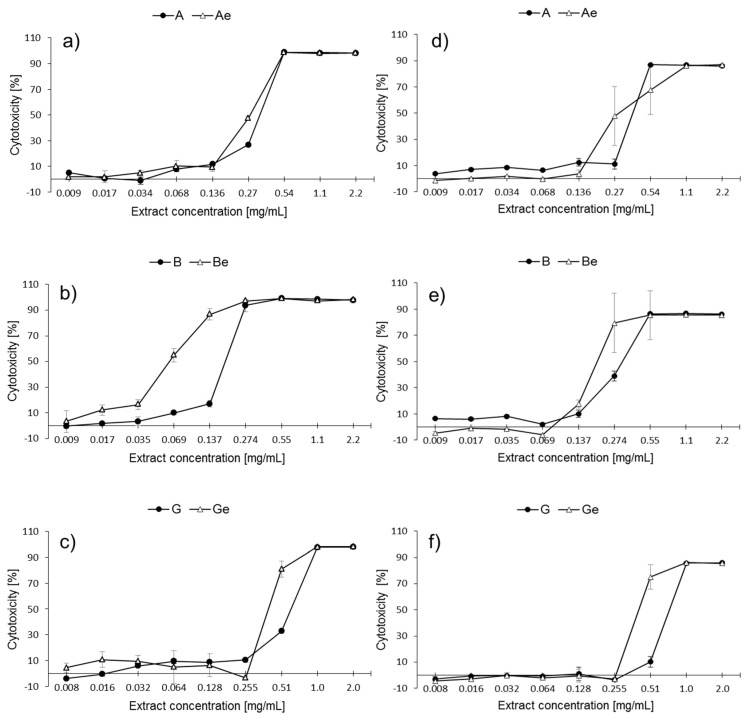
Cytotoxic activity of the *P. quinquefolium* extracts towards Caco-2 cells determined by MTT (**a**, **b**, **c** graphs) and PrestoBlue (**d**, **e**, **f** graphs) assays after 72 h of exposure. Each value represents the mean of four repeats ± SD. Letter *e* in italic indicates extract of plant subjected to elicitation.

The cytotoxic activity of the *P. quinquefolium* extracts towards Caco-2 cells increased together with the rising extract concentration (Figure 2). It could be observed that the highest concentrations of the tested extracts exerted the strongest metabolic inhibitory effect, while the lowest concentrations did not affect the cells significantly.

In the MTT assay, for extract A, the three highest concentrations of MeJa were associated with the greatest decrease in the metabolic activity (up to 98.7% ± 0.2%). The lowest values of cytotoxic effects were observed for concentrations lower than 0.136 mg/mL (Figure 2a). Extract A*e* exerted a similar biological activity towards Caco-2 cells to extract A. In the case of extract B, a significant increase in cytotoxicity was observed between concentrations 0.137 mg/mL and 0.274 mg/mL (from 17.2% ± 2.6% to 93.6% ± 4.8%) (Figure 2b), together with a significant decrease in cell viability. Extract B*e* was found to be the greatest inhibitor of Caco-2 metabolic activity, demonstrating toxic effects from a minimum concentration of 0.035 mg/mL. The four highest concentrations demonstrated the greatest inhibition of cell viability (up to 98.9% ± 0.3%). Extracts G and G*e* demonstrated similar effects (Figure 2c); however, at 0.51 mg/mL, Ge had a stronger effect than G. Extracts G and G*e* only exerted a strong cytotoxic activity (approximately 98%) when administered at the three highest concentrations, as well as the lowest influence on the metabolic activity.

In the PB assay, for extracts A and A*e*, a relevant increase in the cytotoxic activity (from 11.2% ± 3.9% to 86.9% ± 0.3%) was observed between concentrations 0.27 and 0.532 mg/mL. The strongest increase in cytotoxicity was observed between the concentrations of 0.27 and 0.54 mg/mL (Figure 2d). Similar tendencies were observed for extract B and extract B*e*: the strongest cytotoxicity was observed for the highest concentrations (up to approximately 86%). For extracts G and G*e* (Figure 2f), a rapid decrease in the metabolic activity (from 10.3% ± 4.0% to 85.8% ± 0.1% for extract G) was observed, starting from the concentration of 0.51 mg/mL. In the case of G*e*, a strong increase of cytotoxicity up to 75.1% ± 9.3% was observed, starting from the concentration 0.255 mg/mL. The highest concentrations of both extracts were the strongest inhibitors of the metabolic cellular activity (up to approximately 86%). Furthermore, extract G demonstrated the lowest cytotoxic activity among all the studied extracts. Generally, the *P. quinquefolium* extracts derived from the plant cultures that underwent elicitation displayed a stronger influence on cellular viability than those that did not.

### 2.3. Estimation of Half Maximal Inhibitory Concentration (IC_50_)

IC_50_ is defined as the concentration of a compound which is required to reduce cell survival to 50% of the control values. The IC_50_ values of all the *P. quinquefolium* extracts were determined on the basis of MTT and PB assays (Table 2). The IC_50_ value for each extract, calculated based on the results obtained by the MTT and PB assays, were similar. The highest cytotoxic effect was documented for extract B*e* (0.06 in MTT and 0.21 mg/mL for PB). The least cytotoxic appeared to be extract G, reaching an IC_50_ of 0.64 mg/mL (in MTT) and 0.77 mg/mL (in PB). According to the IC_50_ values, the cytotoxicity of the *Panax* extracts ranked as follows: B*e* and B > A*e* and A > G*e* and G. The extracts obtained by elicitation demonstrated lower IC_50_ values than those that were not, indicating that the elicited *P. quinquefolium* plants demonstrate a higher cytotoxic activity. The Presto Blue and MTT assay results indicate comparable patterns of cytotoxicity. The higher sensitivity indicated by the MTT assay may result from the fact that it induces mitochondrial dysfunction, thus augmenting the effect of the extract [24].

In general, our findings indicate that the *P. quinquefolium* hairy root extracts derived from the cultures that underwent MeJA elicitation had stronger cytotoxic properties. The analysis of the IC_50_ values showed that these parameters are lower for the A*e*, B*e* and G*e* clones than for the A, B and G clones, respectively. This would suggest that higher saponin levels are associated with a stronger cytotoxic activity against Caco-2 cells. However, in contrast, extracts B and B*e* were the most cytotoxic, even though they contained the lowest level of ginsenosides. This is strong evidence that the observed biological activity relies on the chemical composition rather than the total quantity of the compounds: the two extracts were the richest sources of Rc, Rb1 and Rg1 ginsenosides. In addition, these findings might be attributed to the presence of rare ginsenosides such as Rh2, Rh3, Rg2 or Rg5, which were not studied in the present study. The literature data indicated that these metabolites demonstrate cytotoxic, anti-cancer and anti-proliferative activities; however, they also appear in greater quantities after subjecting field-cultivated roots to high temperatures [25,26,27,28,29].

A previous study [30] examined the anti-proliferative activity of *P. quinquefolium* extracts towards HCT-116 colorectal cancer cells by the modified trichrome stain (MTS) method. Higher concentrations of the extracts were found to be associated with lowered cell viability. At lower concentrations (0.1–0.25 mg/mL), the anti-proliferative activity was minimal, while a significantly higher (above 90%) activity was observed for the higher concentrations (0.5 mg/mL) [30]. In our case, the pattern of results was similar.

A previous MTT-based study of the cytotoxicity of a *P. quinquefolium* extract towards hepatocellular carcinoma cells (SMMC-7721) also found that the survival rate of cells decreased along with the increase in the extract concentration. The cells were incubated with different extract concentrations (0, 20, 40, 60 and 80 mg/mL) for 12 h. [31]. The Rg3 ginsenoside level was also found to significantly decrease 375.S2 melanoma cell viability compared with controls (IC_50_ 20 μM) [32]. Li et al. [33] observed that the total ginsenoside extract of Chinese ginseng containing a mixture of Rg1, Re, Rd and Rb1 induced stronger cytotoxicity against HT-29 human colon cancer cells than its individual ginsenoside components. After a 72-h treatment, the IC_50_ was equal to 0.105 mg/mL.

### 2.4. Basal Endogenous DNA Damage Induced by P. quinquefolium Extracts

The genotoxicity of the different concentrations of the *P. quinquefolium* extracts was estimated by means of a comet assay. The mean percentage of DNA in the comet tail ± S.E.M. at the different concentrations of the extract is given in Table 3. The choice of concentrations was based on the IC_50_ data analysis (close or lower than IC_50_).

Negative control cells demonstrated 4.2% ± 0.3% DNA damage, while the positive controls demonstrated 40.6% ± 3.6%. The genotoxic activity of extracts was observed to be dose-dependent. The highest concentrations of the *P. quinquefolium* extracts were noticed to be the most genotoxic. The lowest extract concentrations displayed a slightly higher genotoxic activity than the medium ones. Extracts A and A*e* at concentrations 0.017 and 0.068 mg/mL induced comparable results in DNA damage, i.e., up to 10.2% ± 0.6%. At a concentration of 0.27 mg/mL, extract A was found to be 2.5-times more genotoxic than A*e*, resulting in 63.5% ± 1.9% DNA damage compared with 25.6% ± 2.3%. Extracts B and B*e* demonstrated similar genotoxicity at concentrations of 0.009 and 0.035 mg/mL. Extract B*e* seemed to display stronger genotoxic effects at a concentration of 0.137 mg/mL (40.9% ± 2.4%) than extract B (34.0% ± 3.3%). At a concentration of 0.51 mg/mL, extract G demonstrated 66.6% ± 1.8% genotoxicity, while at 0.255 mg/mL G*e* exerted 41.6% ± 2.7% genotoxicity. Extract G*e* was probably more genotoxic than G, indicated by the fact that half the concentration was needed to induce similar genotoxic effects to G. Those values cannot be exactly compared due to the different tested concentrations (chosen on the basis of the IC_50_ values).

No correlation was found between genotoxic effects and the source plant species. There is no data on the genotoxicity of *P. quinquefolium* extracts on cell lines, but Zhang et al. [34] found Rg3 ginsenoside to significantly increase DNA damage in a concentration-dependent manner in human osteosarcoma cells. Rg3 also induced double-strand breaks, which can lead to chromosome aberrations.

### 2.5. Effect of P. quinquefolium Extracts on Colony Formation

**Figure 3:**
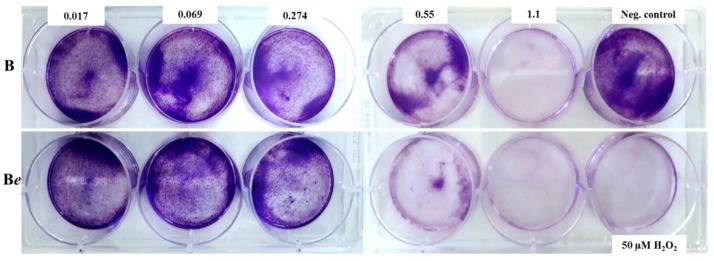
Images representing colonies produced by Caco-2 cells following plating of 1000 cells and 7 days of incubation. Cells were treated with 0.017-1.1 mg/mL concentrations of the *P. quinquefolium* B (non-elicited) and B*e* (elicited) extracts for 60 min. A positive control in a form of 50 µM H_2_O_2_ was used. Cells in negative control were not treated. Letter *e* in italic indicates extract of plant subjected to elicitation.

Extracts B and B*e* displayed the strongest cytotoxic and anti-proliferative effects and so were subjected to further research based on a colony forming assay: the survival and proliferative capacity of cells treated with a cytotoxic agent are measured based on their ability to form colonies. It was found that the pre-treatment of cells with *Panax* B and B*e* extracts effectively inhibited the colony formation (Figure 3), which was clearly visible in the samples containing extracts of concentrations equal to and higher than 0.55 mg/mL. These findings confirm that extracts were not only able to decrease the metabolic activity of Caco-2 cells but also had an anti-proliferative effect. An analysis of the colony formation ability of melanoma A375.S2 cells 24 h after treatment with different Rg3 ginsenoside concentrations found this ability to decrease for all Rg3 concentration levels compared with controls [32].

### 2.6. The Effect of P. quinquefolium Extracts on Intracellular ATP Level, Mitochondrial Membrane Potential, Intracellular Oxidative Stress and Apoptosis Induction

**Figure 4:**
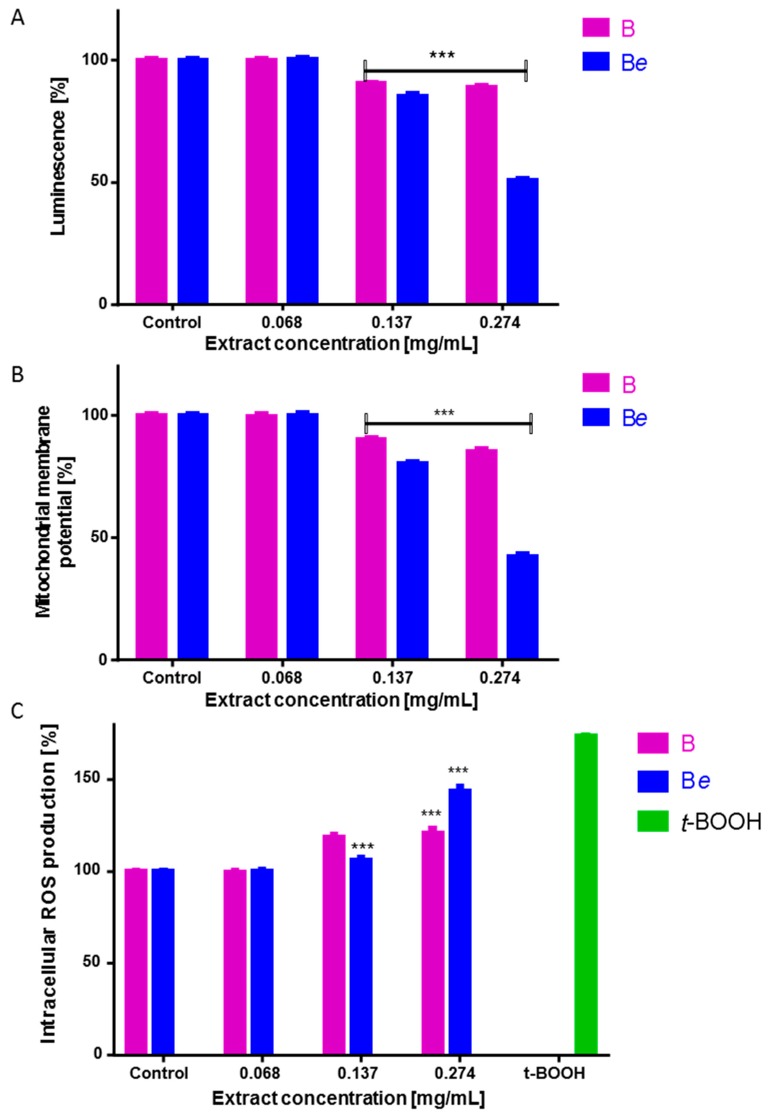
The effects of the 48-h *P. quinquefolium* B (non-elicited) and B*e* (elicited) extract treatment on the ATP level of Caco-2 cells as determined by the ATP luminescent assay kit (**A**); mitochondrial membrane potential was determined with a JC-1 probe (**B**); intracellular reactive oxygen species (ROS) generation was analyzed by a DCFH-DA assay (**C**), as a positive control of 50 µM *t*-BOOH was used; control cells were not exposed to any compound but the vehicle (medium). Values are presented as the mean (n ≥ 8) ± SD; statistical significance was calculated versus the control cells (untreated), *** *p* < 0.001.

Further analyses at concentrations not exceeding the IC_50_ values were performed to determine the molecular mechanism of the B and B*e* ginsenosides’ cytotoxicity against Caco-2 cells. It was found that both extracts influenced cellular ATP production (Figure 4A). The ATP level in Caco-2 cells decreased by 20% following the treatment with 0.137 mg/mL extract of the plant following elicitation; this decreased to 50% at the higher concentration of 0.274 mg/mL. Ginseng B preparation reduced luminescence by 10–15% at all studied dosages.

**Figure 5:**
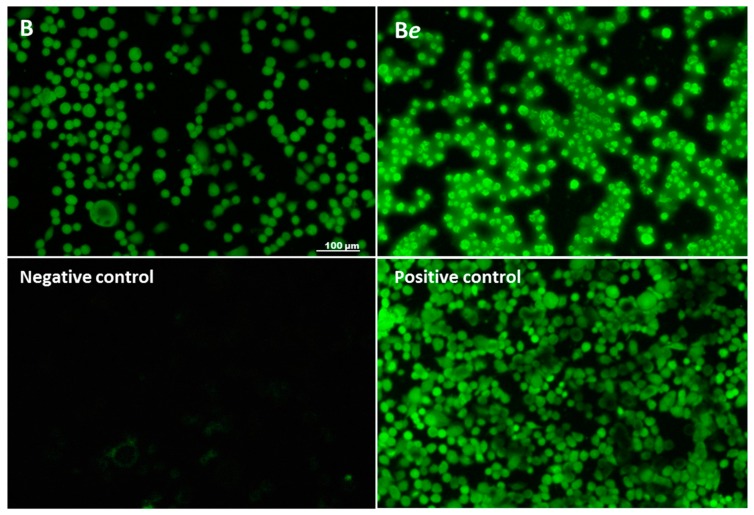
Intracellular ROS generated in Caco-2 cells after staining with DCFH-DA. The *P. quinquefolium* extract (0.274 mg/mL) B non-subjected to elicitation (with lower fluorescence) and B*e* subjected to elicitation (with higher fluorescence). Negative control—healthy cells without fluorescence (no ROS). Positive control (50 µM *t*-BOOH) with highly fluorescent cells. Fluorescence microscopy (Nikon, Tokyo, Japan), 200× magnification.

Both extracts reduced the mitochondrial membrane potential in a concentration-dependent manner (Figure 4B). While extract B diminished the potential by up to 15%, extract Be reduced the value by 20% to 60%. This observed decrease in the mitochondrial potential was accompanied by an intracellular increase in the ROS level for both B and B*e* (Figure 4C); however, 0.137 mg/mL extract B was a stronger inducer of oxidative stress: it elevated fluorescence by nearly 20% compared to controls, whereas 0.274 mg/mL B*e* extract increased the ROS level by nearly 45%. These quantitative results were confirmed by fluorescence microscopy observations: cells treated with extracts B and B*e* demonstrated higher fluorescence than untreated cells due to the higher ROS concentration (Figure 5). Extract Be demonstrated a stronger intracellular ROS accumulation.

**Figure 6:**
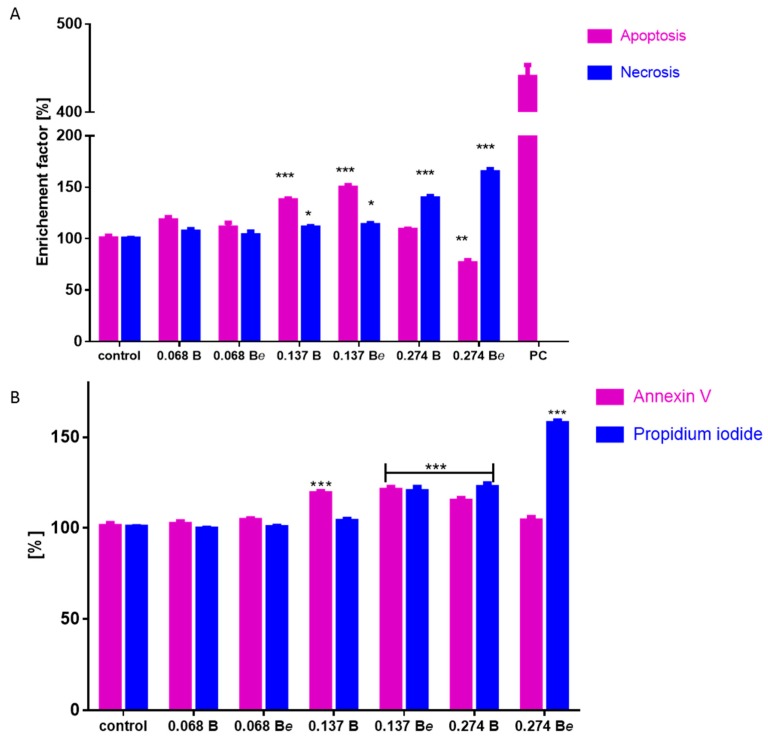
The effects of the *P. quinquefolium* B (non-elicited) and B*e* (elicited) extracts after 48 h treatment of Caco-2 cells: phosphatidylserine externalisation on the outer membrane leaflet of the apoptotic cells and membrane permeabilization were detected with the annexin-V-FITC assay kit and propidium iodide staining (**A**); the late stage of apoptosis was analyzed by a cell death detection kit (**B**), PC—internal positive control of the assay. Control cells were not exposed to any compound but the vehicle (medium). Values are presented as the mean (n ≥ 8) ± SD; statistical significance was calculated versus the control cells (untreated), * *p* < 0.05, ** *p* < 0.01, *** *p* < 0.001.

The observed decrease in the mitochondrial potential and ATP level, as well as the intensive elevation of ROS, indicated that cellular death may be triggered, like apoptosis or necrosis. Therefore, the next part of the study investigated the impact of the ginseng extracts on apoptosis induction by the detection of externalized phosphatidylserine (PS) in the cell membrane using annexin-V-FITC/propidium iodide staining (Figure 6A). Annexin V binds to externalised phosphatidylserine on the outer membrane leaflet of apoptotic cells, whilst the propidium iodide stains the nuclei of cells with perforated membranes. The highest number of apoptotic cells positive for annexin V staining was observed for 0.137 mg/mL of ginseng B (about 18%), whereas high levels of cells positive for both annexin V and propidium iodide were observed for the B*e* extract at 0.137 mg/mL and the B extract at 0.274 mg/mL. Ginseng B*e* at a 0.274 mg/mL concentration revealed a high red fluorescence quantity of cells with stained nuclei (about 58%) specific for necrosis or secondary necrosis of apoptotic bodies not engulfed by neighbouring cells. A subsequent DNA fragmentation analysis of the cytoplasmic mono- and oligonucleosomes revealed a significant increase in apoptosis induction by the B and B*e* extracts at 0.137 mg/mL (Figure 6B). Further investigation showed that cells treated with a 0.274 mg/mL concentration of both preparations showed predominantly necrotic death-type features due to the presence of cell-released nucleosomes in the culture medium.

**Figure 7:**
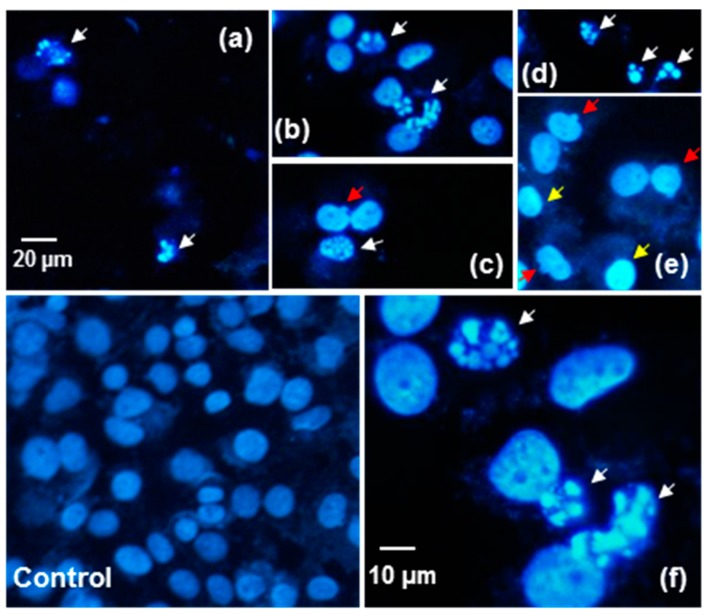
DAPI-stained nuclei of Caco-2 cells after exposition to 0.137 mg/mL of the *P. quinquefolium* extracts B (non-elicited) (**a**–**c**) and B*e* (elicited) (**e**–**f**). Apoptotic bodies (white arrows), chromatin condensation (yellow arrows) and nuclear fragmentation (red arrows). Magnifications 200× (control, **a**–**e**) and 400× (**f,** and magnified **b**).

**Figure 8:**
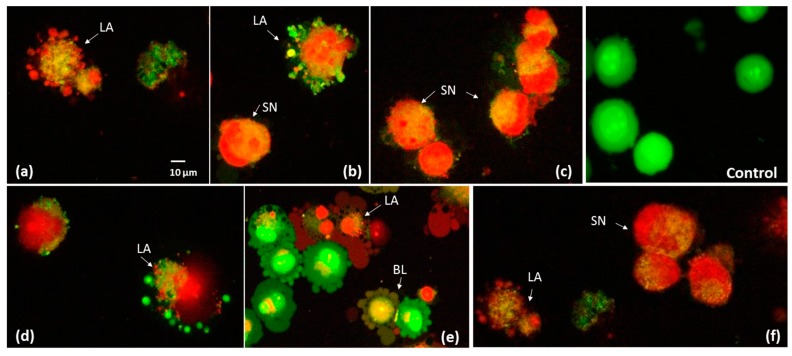
Fluorescent images of AO/PI double staining of Caco-2 cells exposed to 0.137 mg/mL (**a**,**b**,**d**),**)e**) and 0.274 mg/mL (**c**, **f**) of the *P. quinquefolium* extracts B (non-elicited) (**a**–**c**) and B*e* (elicited) (**d**–**f**). LA—late apoptosis, BL—blebbing, SN—secondary necrosis. 400× magnification.

The current results are consistent with the microscopic observations of the cellular morphology changes occurring after cellular death induction. DAPI staining allows morphological changes in cell nuclei to be assessed. The nuclear morphology of Caco-2 cells was evaluated after 48 h of exposure to 0.137 mg/mL of the B and B*e* extracts. Numerous apoptotic bodies, chromatin condensation and nuclear fragmentation could be observed (Figure 7). AO/PI staining analyses were also conducted according to the criteria given by Baskić et al., 2006 [35] and Salim et al., 2013 [36]. The control cells (viable) exhibited a green fluorescence with a light-green nucleus with an intact structure of the chromatin (Figure 8). An orange colour, chromatin fragmentation, cell shrinkage and cell membrane blebbing were symptoms of late apoptosis, while bright-red nuclei with condensed chromatin indicated direct necrosis. Kim et al., 2019 [32] demonstrated that the Rg3 ginsenoside induced apoptosis in A375.S2 melanoma cells related to the mitogen-activated protein kinase signalling pathway. The authors also observed morphological changes in cells such as membrane blebbing. Li et al., 2018 [33], in DAPI staining, observed nuclear changes in the colon cancer cell HT-29 typical for apoptosis, such as karyopyknosis, chromatin condensation and nuclear fragmentation. These observations were made for the total ginsenosides of Chinese ginseng containing Rg1, Re, Rd and Rb1.

There are many reports indicating that Rg3, Rh2, Rg5, Rk1 and Rh4 ginsenosides act as apoptosis inducers in different types of cell lines [37,38,39]. It is supposed that the most potent apoptosis activators among saponins are those with less polar chemical structures [40]. Recently, it was demonstrated that not only ginsenosides but also their metabolites secreted by intestinal bacteria, like compound K, are able to activate apoptosis via the induction of intracellular reactive oxygen species and mitochondria membrane potential loss [41]. Remarkably, apoptosis as a programmed cell death (implicated in the removal of defective or unwanted cells without inflammation induction) is one of the tools used in cancer prevention. Studies performed on a BALB/c nude mouse model of human breast cancer demonstrated that Rg5 activates caspase-dependent apoptosis via the activation of the extrinsic death receptor and intrinsic mitochondrial signalling pathways [42]. We examined the ginsenoside involvement in Caco-2 cell death induction via oxidative stress generation; however, a more detailed evaluation of specific markers connected with cellular death, such as caspases−3/−9 activation or the appearance of specific proteins, i.e., t-Bid, cytochrome c, Bax and Bak, is required [40]. Such a molecular identification is very important because in human colorectal cancer HCT116 cells, there have been demonstrated studies identifying ginsenosides Rh2 and Rg3 as not only inducers of apoptotic-type cellular death but also as activators of paraptosis [43]. That type of cell death is independent of caspase activation and is characterized by cytoplasmic vacuole formation, mitochondrial swelling and clumping.

The present study also examines the ability of the extracts to induce necrosis. However, it is important to mention that some proteins involved in the intrinsically regulated type of cell death, which shares features of apoptosis and necrosis, are responsible for the induction of the cellular death type known as necroptosis [44]. On the other hand, the treatment of H9c2 cardiomyocytes with the deglycosylated ginsenoside compound Mc1 significantly increased the levels of catalase and superoxide dismutase and reduced the elevation of the proapoptotic Bax/Bcl2 ratio and caspase-3 activity [45]. Due to these facts, the identification of the detailed mechanism of the biological activity of *P. quinquefolium* ginsenosides requires further investigation.

## 3. Materials and Methods

### 3.1. Chemicals and Reagents

The *N*-2-hydroxyethylpiperazine-*N’*-2-ethanesulfonic acid (HEPES) buffer, Dulbecco’s modified Eagle’s medium (DMEM), streptomycin/penicillin mixture, phosphate buffered saline (PBS, pH 7.2), trypan blue dye, 3-(4,5-dimethylthiazol-2-yl)-2,5-diphenyltetrazolium bromide (MTT), dimethyl sulfoxide (DMSO), low melting point (LMP) agarose, normal melting point (NMP) agarose, NaCl, Triton X-100, EDTA, Tris, NaOH, paraformaldehyde, 4,6-diamidino-2-phenylindole (DAPI), acridine orange (AO), propidium iodide (PI), 2′,7′-dichlorofluorescin diacetate (DCFH-DA), hydrogen peroxide (H_2_O_2_), annexin-V-FITC assay kit, *tert*-butyl hydroperoxide (*t*-BOOH), and methyl jasmonate (MeJA) were derived from Sigma-Aldrich (St. Louis, MO, USA). The Rb1, Rb2, Rb3, Rc, Rd, Re, Rg1 and Rg2 ginsenosides standards were purchased from C. Roth GmbH + Co Karlsruhe, Germany. The foetal bovine serum (FBS), GlutaMAX^TM^, TrypLE^TM^ Express, PrestoBlue (PB), 5,5′,6,6′-tetrachloro-1,1′,3,3′-tetraethyl-imidacarbocyanine iodide (JC-1) originated from Invitrogen Thermo Fisher Scientific (Waltham, MA, USA). The human colon adenocarcinoma cell line Caco-2 from the 50^th^ passage was purchased from Cell Line Service GmbH (Eppelheim, Germany). The 0.20 and 0.22 μm pore size syringe filters were from Merck Millipore (Darmstadt, Germany). The Cell Death Detection ELISA^Plus^ was purchased from Roche Diagnostics (Basel, Switzerland). The CellTiter-Glo^®^ Luminescent Cell Viability Assay kit was from Promega (Madison, WI, USA).

### 3.2. P. quinquefolium Hairy Root Culture

Three clones (A, B and G) of the *P. quinquefolium* hairy root cultures were grown in 300 mL shake Erlenmeyer flasks with 80 mL of modified, hormone-free B-5 medium [14]. The cultures were maintained in the dark at 26 °C degrees on a rotary shaker (100 rpm). The extracts were prepared from three different clones of the transformed roots of *P. quinquefolium* and were used for the biological assays. They were derived from cultures that did not undergo an elicitation process (A, B and G) and from the hairy roots that were elicited by the 250 µM MeJA (A*e*, B*e* and G*e*). A stock solution containing 95% MeJA in 96% ethanol (sterilized through a Millipore filter of pore size 0.20 μm) was added to the medium on the 25th day of culture. The ginseng saponins accumulation in the hairy root cultures of *P. quinquefolium* was examined after seven days of the MeJA treatment.

### 3.3. Preparation of P. quinquefolium Roots’ Extracts

The hairy root cultures, after 32 days of growth in in vitro conditions, were rinsed under running water to remove medium residue, dried at room temperature and subjected to extraction in 80% methanol and solid phase extraction, as described earlier [14]. The dried hydromethanolic extracts, taken from all the tested cultures, were weighed and used for the quantitative analysis of the ginsenosides (HPLC method). For further investigations of the biological activity towards Caco-2 cells, the stock solutions were prepared after dissolving in a complete culture medium for Caco-2 cells (without phenol red). They were sterile-filtered (0.22 μm pore size) and diluted to the 10× stock concentrations from 0.08 to 22 mg/mL. The stocks were stored at −20 °C.

### 3.4. Determination of Ginsenoside Content Using HPLC Method

The samples were examined for the presence of eight ginsenosides (Rb1, Rb2, Rb3, Rc, Rd, Re, Rg1 and Rg2) using a liquid chromatography system consisting of an Agilent Technology 1200 apparatus, a ZORBAX Eclipse XDB-C18 (150 × 4.6 mm, 5 µm) column, Quat Pump and UV–Vis DAD type detector, as well as an Agilent Technology set combined with the Agilent ChemStation 2001–2010 software. The details of this analysis are presented in our earlier report [46]. The ginsenoside content was expressed as milligrams per gram of dry weight.

### 3.5. Caco-2 Cell Culture

Caco-2 cells were maintained according to Nowak et al., 2017 [47]. They were cultured in DMEM, supplemented with 10% FBS, 4 mM GlutaMAX^TM^, 25 mM HEPES buffer and 100 µg/mL streptomycin/100 IU/mL penicillin mixture for 7 days at 37 °C in the atmosphere of 5% CO_2_. Every 2–3 days, the cells were washed with PBS and supplemented with a fresh medium. Confluent cells were detached with TrypLE^TM^ Express. The cell suspension was centrifuged (182× *g*, 5 min), decanted and the pellet was re-suspended in fresh DMEM. After the determination of the cell count by haemocytometer and cell viability by trypan blue exclusion, the Caco-2 cells were ready to use.

### 3.6. MTT and PB Assays

In each well of a 96-well plate, 1 × 10^4^ Caco-2 cells were seeded in a complete culture medium and incubated overnight (37 °C, 5% CO_2_). Next, the medium was aspirated, and the plant extracts were added to achieve final concentrations of from 0.008 to 2.2 mg/mL. The negative control contained cells in DMEM. Cells were exposed to extracts for 72 h (37 °C, 5% CO_2_). After incubation, the samples were aspirated; MTT (0.5 mg/mL) was added and the samples were further incubated for 3 h. Next, the dye was removed, and formazan precipitates were solubilised by DMSO. Absorbance was measured at 550 nm (with a reference filter of 620 nm) using a microplate reader (TriStar² LB 942, Berthold Technologies GmbH & Co. KG, Bad Wildbad, Germany).

In the case of the PB assay, after removing the test samples, the PB reagent (10%) was added to each well and the samples were incubated at 37 °C under 5% CO_2_ for 2 h. The fluorescence signal at F560/590 nm was then measured, using a microplate reader. Both experiments were conducted with the same cell’s population.

The absorbance/fluorescence of the control sample (untreated cells) represented 100% cell viability. Cell viability (%) was calculated as [sample OD (optical density) or fluorescence/control OD or fluorescence] × 100%; and anti-proliferative activity/cytotoxicity (%) as 100–cell viability. Results were presented as the mean ± standard deviation (SD)/standard error of the mean (S.E.M.). The IC_50_ value was used as a measure of cellular sensitivity towards a given treatment and was determined by MTT and PB assays according to OECD (The Organisation for Economic Co-operation and Development) protocol, 2015 [48], according to the following formula: IC_50_ = (X − Z)/(X − X_1_) × (CX_1_ − CX) + CX, where X is a 50% decrease in viability; X is the % of viability > Z; X_1_ is the % viability < Z; CX is the concentration of the compound for X, and CX_1_ is the concentration of the compound for X_1_.

### 3.7. Genotoxicity Testing (Comet Assay)

The concentrations of the extracts for genotoxicity testing were selected on the basis of the cytotoxicity screening and IC_50_ values. The cells were incubated (37 °C, 1 h) in a supplement-free medium with the following concentrations of the *P. quinquefolium* extracts [mg/mL]: A) 0.017; 0.066 and 0.226; A*e*) 0.017; 0.069 and 0.275; B) 0.008; 0.034 and 0.135; B*e*) 0.009; 0.035 and 0.139; G) 0.032; 0.126 and 0.504; G*e*) 0.016; 0.065 and 0.258. The Caco-2 cells’ final concentrations were adjusted to 10^5^ cells/mL in each sample. The negative control consisted of Caco-2 cells in DMEM, while the positive control contained 50 µM H_2_O_2_. The final amount of each sample was set to 1 mL. The comet assay was performed under alkaline conditions (pH > 13) as previously described [47]. After incubation, aliquots of suspended cells were centrifuged (182× *g*, 15 min, 4 °C), decanted, suspended in 0.75% LMP agarose and distributed onto slides precoated with 0.5% NMP agarose and immersed in a lysing solution consisting of 2.5 M NaCl, 1% Triton X-100, 100 mM EDTA and 10 mM Tris, with pH 10 (4 °C, 1 h). After the lysis, the slides were subjected to horizontal gel electrophoresis and the DNA was allowed to unwind for 20 min in an electrophoretic solution, containing 300 mM NaOH and 1 mM EDTA. Electrophoresis was conducted at 4 °C for 30 min at an electric field of strength 0.73 V/cm (300 mA). Then, the slides were neutralised with distilled water for 5 min, stained with 1 mg/mL PI and covered with cover slips. The objects were visualised at 200× total magnification in a fluorescence microscope (Nikon Eclipse Ci H600L, Tokyo, Japan), attached to a digital camera (Nikon Digital Sight DS-U3, Tokyo, Japan) and connected to a Lucia-Comet v. 7.0 PC-based image analysis system (Laboratory Imaging, Prague, Czech Republic). One hundred images were randomly selected from each sample and the percentage of DNA in the comet tail was measured. The results were presented as the mean ± standard error of the mean (S.E.M.).

### 3.8. Clonogenic Assay

As extracts B and B*e* appeared to display the strongest cytotoxic and anti-proliferative effects, they were thus taken for further analysis according to Choi et al., 2018 [49], with some modifications. To each well of a 6-well plate, 3.5 × 10^5^ cells were seeded and cultured to reach 80% confluence. After that, cells were washed with PBS and exposed to extracts of concentrations from 0.017 to 1.1 mg/mL for 60 min. The positive control was 50 µM H_2_O_2_. After that, all cells in each well were harvested, and counted according to the standard procedures. Next, 1000 cells were inoculated on each well of the 6-well plate and cultured for 7 days to enable the formation of the colonies. The colonies were fixed with 3.7% paraformaldehyde for 15 min, air-dried and stained with 0.1% crystal violet.

### 3.9. Measurement of ATP Production and Mitochondrial Membrane Potential (MMP)

The intracellular ATP level was quantified with a CellTiter-Glo^®^ Luminescent Cell Viability Assay kit according to the manufacturer’s instructions. Briefly, the cells were incubated with the compounds for 48 h, following which, the single reagent was added directly to the cells. After the cell lysis, the luminescence was measured—this was proportional to the amount of ATP present. The measurements were performed using the Synergy 2 BioTek Microplate Reader and calculated according to the formula:

Luminescence [%] = luminescence of the sample cells/luminescence for the control cells × 100(1)

The MMP was assayed with the JC-1 probe. After 48 h treatment with the studied compounds, the medium was changed and JC-1 (1 μg/mL) was applied for 20 min. Then, the cells were washed with a serum-free medium and the fluorescent signals at F485/530 and F530/620 nm were measured and the ratio of the obtained values F485/530 and F530/620 nm were taken for the calculation according to the formula:

Mitochondrial membrane potential [%] = (ratio of F_530/620_ and F_485/530_ the sample cells/ratio of F_530/620_ and F_485/530_ for the control cells) × 100 (2)

### 3.10. Detection of Intracellular Reactive Oxygen Species (ROS) Generation

To determine the effect of extracts on the intracellular generation of ROS after the 48-h treatment, cells were loaded with the DCFH-DA dye at a final concentration of 10 μM for 30 min. The fluorescent signal was analyzed at a wavelength of F485/530 nm. Calculations were performed according to the formula:

Intracellular ROS production [%] = fluorescence of the sample cells/fluorescence of the control cells × 100(3)

For the microscopic observations, the experiment was conducted in 8-well Lab-Tek™ Chamber Slides. The negative control contained only cells in DMEM (without FBS), while the positive control contained 50 µM *t*-BOOH. The intracellular fluorescence of cells was observed under a fluorescence microscope after 6 h of treatment. An increased intensity of intracellular fluorescence was an indication of an increased level of the generated ROS.

### 3.11. Phosphatidylserine Externalisation and Membrane Permeabilization

After 48 h treatment, the cells were washed twice with PBS and incubated with annexin-V-FITC (final concentration 0.25 μg/mL) for 10 min. Annexin-V binding was measured by the change in fluorescence (F485/530 nm). Membrane permeabilization caused by the investigated compounds was measured using propidium iodide (PI). After 48 h treatment of the cells, PI was added at a final concentration of 1 µg/mL. Intercalation was monitored by the change of fluorescence F535/620 nm. For each of the parameters studied calculations were performed according to the formula:

[%] = fluorescence of the sample cells/fluorescence of the control cells × 100(4)

### 3.12. Detection of Mono- and Oligonucleosomes Release (Apoptotic DNA Degradation and Necrosis Detection)

The late stage of apoptosis was measured by Cell Death Detection ELISA Plus according to the manufacturer’s instructions. After 48 h treatment, the cells were lysed and the levels of histone-complexed DNA fragments (mono- and oligonucleosomes) present in the cytoplasmic fraction were quantified with an immunoreagent complex. The DNA–histone complex served as the positive control (PC). Following the incubation and washes, the colorimetric solution was added and after adding the stop solution, the colorimetric signal was measured at 405 and 490 nm. The calculation of the enrichment factor of the mono- and oligonucleosomes released into the cytoplasm was performed according to the formula:

Enrichment factor [%] = absorbance of the sample cells/absorbance of the control cells × 100(5)

In order to detect necrosis after the cells’ incubation with compounds, the medium was collected and the level of DNA fragments released from the necrotic cells was determined analogously to the apoptosis measurement.

### 3.13. Fluorescent Microscopic Analysis

For the DAPI staining, each well of a 8-well Lab-Tek™ Chamber Slide was seeded with Caco-2 cells (1 × 10^4^cells/well); for the AO/PI double staining, a 6-well plate was used and each well was seeded with 5 × 10^4^ cells/well and incubated for 24 h to allow them to attach. The tested concentrations of the *P. quinquefolium* extracts B and B*e* were 0.137 and 0.274 mg/mL. After 48 h exposure, the medium with the extracts was gently aspirated. For the AO/PI staining, cells were detached, centrifuged (182× *g*, 5 min), decanted and the pellet was stained with the AO (100 μg/mL) and PI (100 μg/mL) mixture (1:1, *v/v*). The morphology of Caco-2 cells was immediately analyzed under a fluorescent microscope with an imaging software (NIS-elements BR 3.0, Nikon, Tokyo, Japan). In the case of DAPI, after treatment, the cells were washed with PBS, fixed with 3.7% paraformaldehyde for 15 min at room temperature and air-dried. The cells were then stained with 1 μg/mL of DAPI for 5 min, at an ambient temperature in the dark and observed under the microscope.

### 3.14. Statistical Analysis

All data were presented as the mean (n ≥ 4 or 8) ± standard deviation (SD) or standard error of the mean (S.E.M.). All obtained results were subjected to a statistical analysis. The determination comprised the average values and a one-way ANOVA that was followed by the Dunnett’s test using the GraphPad prism 4.0 software (GraphPad Software, Inc. La Jolla, CA, USA) or the OriginPro 6.1 software (OriginLab Corporation, Northampton, MA, USA) at the significance level of * *p* ≤ 0.05.

## 4. Conclusions

The studied clones of the *P. quinquefolium* hairy roots were characterized by different ginsenoside profiles and different contents of individual compounds; higher levels of the studied metabolites were observed in the cultures treated with methyl jasmonate. The use of this elicitor significantly stimulated the accumulation of saponins from the Rb group (increase: 2.4-fold, 4.15-fold and 4.7-fold respectively in the A, B and G clones). The effect of methyl jasmonate on the Rg group saponins was ambiguous: a weak stimulant effect was observed for the B and G clones and an inhibitory effect for clone A.

**Figure 9:**
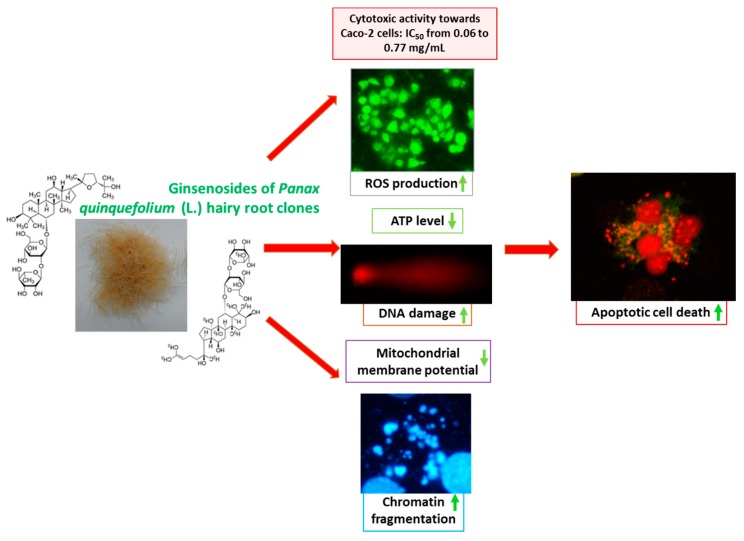
*P. quinquefolium* ginsenosides as agents able to induce apoptosis in Caco-2 cells—proposed mechanism of action.

Here, we also demonstrated the biological activities of the extracts obtained from the *P. quinquefolium* roots (Figure 9). As intestinal cells are mostly influenced by large quantities of dietary and plant-originated compounds, the human epithelial adenocarcinoma Caco-2 cell line was chosen as a cellular model. The main aim of our study was to determine the potential for ginsenosides to induce death in cancer cells, i.e., immortalised Caco-2 cells. Importantly, an apoptosis induction was detected at relatively high dosages. Our findings suggest that extracts derived from the elicited *P. quinquefolium* roots exerted a higher biological activity towards Caco-2 cells than the non-elicited extracts. The elicitation process was associated with significant increases in the Rb group saponins levels in the root cultures. Among the six studied clones of the *P. quinquefolium* hairy root extracts, the strongest inhibitors of the cellular metabolic activity, and apoptosis inducers, were extracts B and B*e* (elicited). Due to the observed depletion of the mitochondrial membrane potential and ATP level, we suspect that the main inducer of apoptosis was the ROS generation. In this case, extracts with high quantities of ginseng demonstrated a prooxidative activity against cells, probably leading to lipid and protein peroxidation, as well as DNA damage.

Moreover, Caco-2 cells are also used as a model of artificial intestine, as they maintain part of the functional capacity of the epithelium in vitro, and are commonly used for in vitro studies of the mechanism of intestinal absorption, and of the cytoprotection against oxidative stress or DNA and proteins damage. It is known that *P. quinquefolium* phytocompounds are able decrease intracellular oxidative stress via chemical radical scavenging, or the recovery and activation of intracellular enzymatic defence, i.e., (glutathione peroxidase (GPx) or superoxide dismutase (SOD) [3]. We are aware that the presented study lacks the presentation of the cytoprotective potential of the obtained ginsenosides. The influence of the obtained extracts as cytoprotective agents and regulators of cellular signalling at non-cytotoxic concentrations will be examined in more detail in further studies. In summary, our findings indicate that further research concerning *P. quinquefolium* hairy root cultures should focus on the examination of rare ginsenosides and other biologically active compound profiles, i.e., phenolic compounds, in order to fully explain the biological properties of *P. quinquefolium* hairy root cultures.

## Figures and Tables

**Table 1:** Ginsenoside content in the studied clones of the *P. quinquefolium* hairy root cultures non-subjected and subjected to elicitation with 250 µM MeJa.

Metabolite		Saponin Content [mg/g d.w.] ± S.E.M.					
		A	B	G	A*e*	B*e*	G*e*
**Rg group**	Rg1	1.28 ± 0.113 ^a^	1.04 ± 0.064 ^a^	1.42 ± 0.037 ^a^	0.79 ± 0.053 ^b^	1.41 ± 0.038 ^a^	2.00 ± 0.063 ^c^
	Re	2.57 ± 0.253 ^a^	0.86 ± 0.037 ^b^	1.27 ± 0.035 ^c^	1.49 ± 0.030 ^d^	1.22 ± 0.021 ^c^	1.21 ± 0.028 ^c^
	Sum	3.85 ± 0.359 ^a^	1.9 ± 0.101 ^b^	2.68 ± 0.072 ^c^	2.28 ± 0.083 ^b^	2.63 ± 0.048 ^c^	3.21 ± 0.089 ^a^
**Rb group**	Rb1	4.74 ± 0.162 ^a^	1.56 ± 0.021 ^b^	1.64 ± 0.034 ^b^	9.91 ± 0.200 ^c^	8.48 ± 0.061 ^d^	9.10 ± 0.282 ^c,d^
	Rc	4.51 ± 0.150 ^a^	1.63 ± 0.194 ^b^	1.35 ± 0.016 ^b^	6.35 ± 0.080 ^c^	2.67 ± 0.041 ^d^	1.83 ± 0.086 ^b^
	Rb2	1.28 ± 0.039 ^a^	0.34 ± 0.014 ^b^	0.22 ± 0.005 ^c^	3.13 ± 0.001 ^d^	0.96 ± 0.038 ^e^	1.10 ± 0.042 ^a,e^
	Rb3	0.76 ± 0.022 ^a^	0.14 ± 0.014 ^b^	0.08 ± 0.002 ^c^	1.83 ± 0.011 ^d^	0.48 ± 0.020 ^e^	0.49 ± 0.018 ^e^
	Rd	1.88 ± 0.114 ^a^	0.56 ± 0.094 ^b^	0.31 ± 0.012 ^b^	10.81 ± 0.141 ^c^	4.94 ± 0.085 ^d^	5.11 ± 0.189 ^d^
	Sum	13.19 ± 0.311 ^a^	4.22 ± 0.245 ^b^	3.72 ± 0.046 ^b^	31.99 ± 0.194 ^c^	17.53 ± 0.217 ^d^	17.64 ± 0.611 ^d^
**Total**	**Rg + Rb group**	**17.04 ± 0.646 ^a^**	**6.12 ± 0.194 ^b^**	**6.27 ± 0.112 ^b^**	**34.96 ± 0.278 ^c^**	**20.16 ± 0.240 ^d^**	**20.85 ± 0.603 ^d^**

The means with the same letter in the row does not differ significantly according to the Kruskall–Wallis test (*p* ≤ 0.05).

**Table 2:** The IC_50_ values of the *P. quinquefolium* extracts determined in MTT and PrestoBlue assays. Letter *e* in italic indicates extract of plant subjected to elicitation.

Extract	IC_50_ [mg/mL]	
	MTT	PrestoBlue
A	0.35	0.40
A*e*	0.29	0.31
B	0.19	0.33
B*e*	0.06	0.21
G	0.64	0.77
G*e*	0.42	0.43

**Table 3:** DNA damage in Caco-2 cells exposed to the *P. quinquefolium* extracts expressed as the mean DNA content in the tail of comets (± S.E.M.) in the alkaline comet assay. The number of cells analyzed was equal to 100. Different letters (a–r) indicate significant differences between results, ANOVA (*p* < 0.05). Letter *e* in italic indicates extract of plant subjected to elicitation.

Extract	Concentration [mg/mL]	DNA in the Tail [%] ± S.E.M.
A	0.017 0.068 0.270	10.2 ± 0.6 ^a,b^ 6.6 ± 1.1 ^a,c^ 63.5 ± 1.9 ^b,c^
A*e*	0.017 0.068 0.270	8.1 ±1.4 ^j^ 9.5 ± 1.5 ^k^ 25.6 ± 2.3 ^j,k^
B	0.009 0.035 0.137	10.2 ± 0.7 ^e,f^ 7.6 ± 1.1 ^e,g^ 34.0 ± 3.3 ^f,g^
B*e*	0.009 0.035 0.137	9.9 ± 0.6 ^l,m^ 7.6 ± 0.8 ^l,n^ 40.9 ± 2.4 ^m^
G	0.032 0.128 0.510	7.6 ± 0.7 ^h^ 8.5 ± 1.7 ^i^ 66.6 ± 1.8 ^h,i^
G*e*	0.016 0.064 0.255	12.1 ± 0.7 ^o,q^ 8.4 ± 1.3 ^o,r^ 41.6 ± 2.7 ^q,r^

## Supplementary Materials

The following is available online, Figure S1: The percentage of individual saponins in relation to the total ginsenoside content in hairy root cultures untreated and treated with MeJa.
