# Supplementary material for: Panax quinquefolium L. Ginsenosides from Hairy Root Cultures and Their Clones Exert Cytotoxic, Genotoxic and Pro-Apoptotic Activity towards Human Colon Adenocarcinoma Cell Line Caco-2
Source: Molecules. 2020 May 11;25(9):2262. doi: 10.3390/molecules25092262 (PMC7249024; doi:10.3390/molecules25092262)
Supplement: Supplementary file 1 [file molecules-25-02262-s001.pdf]

Supplementary Materials

Panax Quinquefolium L. Ginsenosides from Hairy Root Cultures and Their Clones Exert Cytotoxic, Genotoxic and Pro-apoptotic Activity Towards Human Colon Adenocarcinoma Cell Line Caco-2

Ewa Kochan [1,*], Adriana Nowak [2,*], Małgorzata Zakłos-Szyda [3], Daria Szczuka [2], Grażyna Szymańska [1] and Ilona Motyl [2]

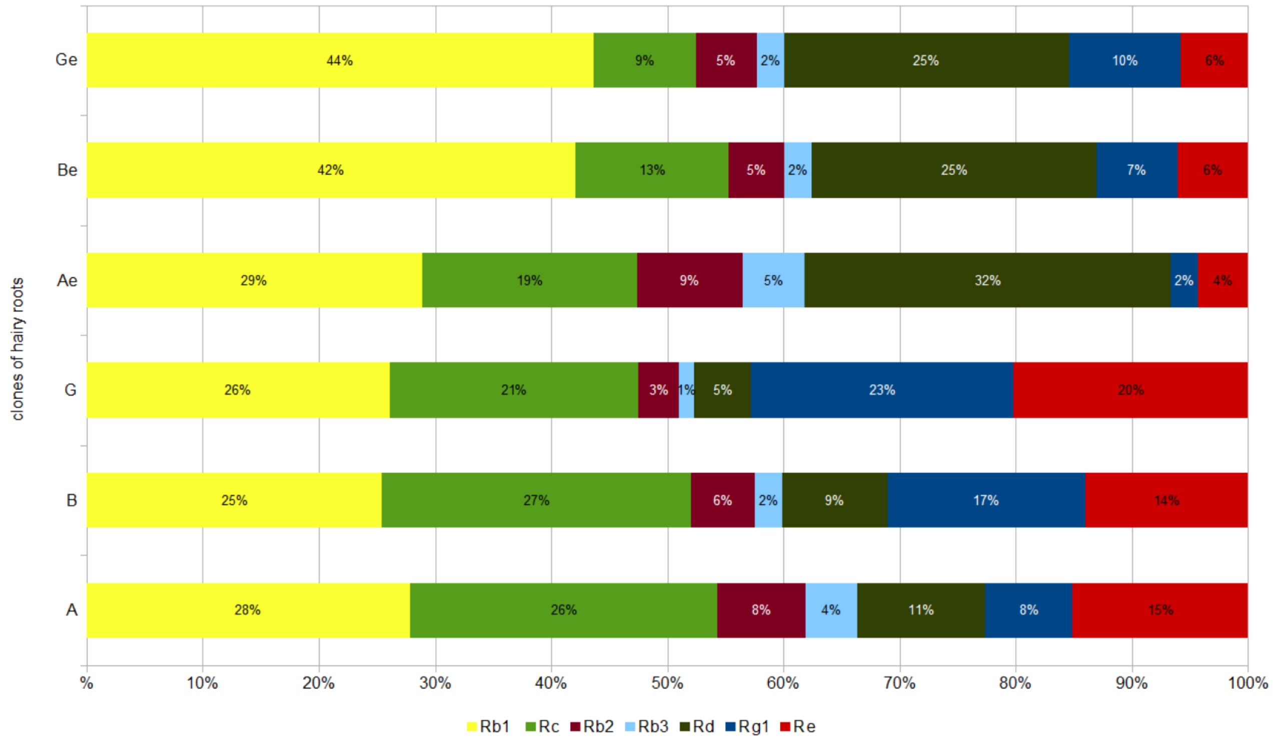

Figure S1. The percentage of individual saponins in relation to the total ginsenoside content in hairy root cultures untreated and treated with MeJa.
